# Supplementary material for: A prospective observational study of community-acquired bacterial bloodstream infections in Metro Manila, the Philippines
Source: PLoS Negl Trop Dis. 2022 May 25;16(5):e0010414. doi: 10.1371/journal.pntd.0010414 (PMC9173634; doi:10.1371/journal.pntd.0010414)
Supplement: S1 File — This supporting material includes additional study protocol, laboratory method, characteristics of control dengue patients, other supporting data. (PDF) [file pntd.0010414.s001.pdf]

Supplementary appendix

This appendix formed part of the original submission and supplied by the authors.

Study Title: "A prospective observational study of community-acquired bacterial bloodstream infections in Metro Manila, the Philippines"

Running title: Community-acquired bacterial bloodstream infections in Manila

Contents:

Appendix 1.
Sample collection and diagnostic tests to be perform per protocol

Appendix 2.
Table S1. Final laboratory and clinical confirmed diagnosis when more than two pathogens were identified by study tests

Appendix 3.
Table S2. Characteristics of patients enrolled as the dengue control (Clinical diagnosed with dengue fever)

Appendix 4.
Table S3. Physical and clinical signs of enrolled patients and the association with positive results of blood culture

Appendix 5.
Table S4. The blood culture volume and blood culture positivity

Appendix 1.

Sample collection and diagnostic tests to be perform per protocol

Samples collected by the study

We obtained blood samples (Acute and Convalescent) from enrolled patients during the enrollment. The blood volume is shown below according to the age.

Acute phase sample

Adult and children over 6 years:

Total 10−19 ml (6-14 ml for bacterial culture divided equally between two blood culture bottles; 1 ml for complete blood count (CBC), 2 mL for renal function and liver enzymes; 2 mL EDTA research sample for polymerase chain reaction (PCR) and other tests.)

For children aged between 3 and 6 years:

Total 8−15 ml (4−10 ml for bacterial culture divided equally between two blood culture bottles (or in one blood culture bottle if volume < 6 mL); 1 ml for CBC, 2 mL for renal function and liver enzymes; 2 mL EDTA research sample for PCR and other tests.

For children aged between 1 and 2 years:

Total 6−11 ml: 2−6 ml for bacterial culture in one blood culture bottle, 1 ml for CBC, 2 mL for renal function and liver enzymes; 2 mL EDTA research sample for PCR and other tests.

Convalescent samples

We obtained an additional 2 mL EDTA research blood sample drawn for convalescent serology between 7 and 10 days of after enrollment or on the day of discharge if sooner.

The research blood sample were taken into an EDTA tube and centrifuged within 3 hours at1000 g for 10 minutes. Then it was divided into buffy coat and plasma in separate tubes. Plasma was divided into two tubes. These were stored at –80°C freezer.

A urine sample was also collected from each patient for microscopy and culture when relevant; an assay of antimicrobial activity; and storage at –80°C

DNA from blood samples

One plasma tube was centrifuged at 21,000 g for 10 min. Then DNA extracted from the plasma pellet using QIAamp DNA Mini Kit (QIAGEN Inc., Valencia, CA, USA). DNA was also extracted from buffy coat samples using the QIAamp DNA Mini Kit (QIAGEN Inc., Valencia, CA, USA).

Diagnostic test to be performed in this study

A set of reference diagnostic tests were performed for each patient according to clinical presentation and the flow was shown in Figure1.

Figure S1: Flow of diagnostic tests to be performed in this study

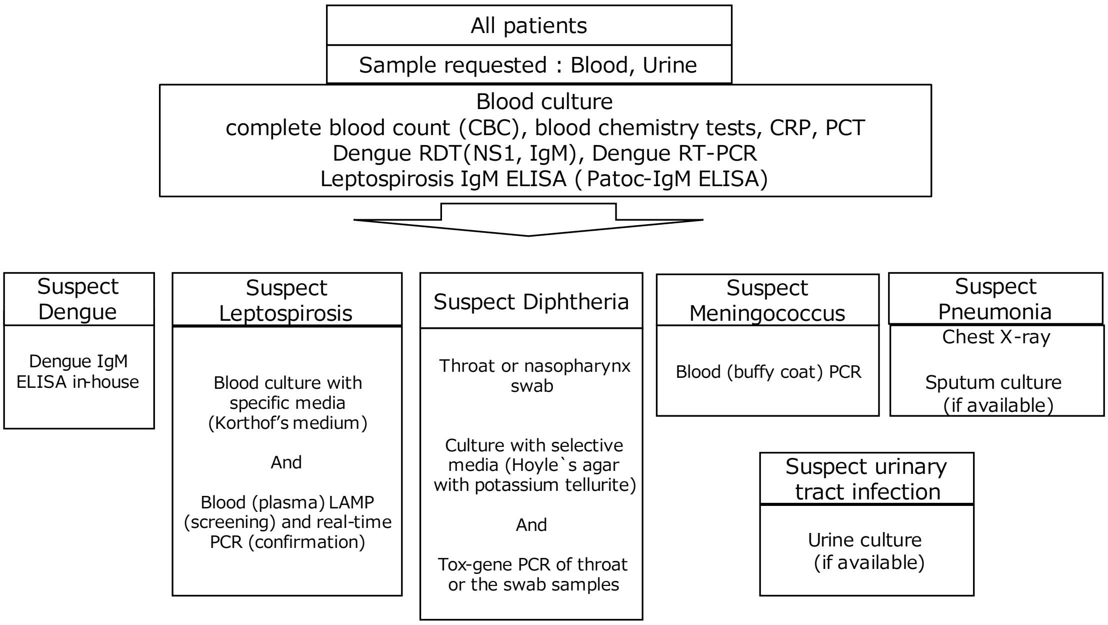

Diagnostic tests to be performed in every case

Complete blood count

Blood culture

Dengue Rapid diagnostic test (RDT) (Dengue Duo, NS1, IgM and IgG, Standard Diagnostics, South Korea).

Dengue reverse transcription-polymerase chain reaction (RT-PCR)

Rapidchip PCT kit (Sekisui Medical, Japan)

Alere Afinion CRP kit with Alere Afinion AS100 Analyzer (Alere Medical, Japan)

Patoc-IgM enzyme linked immunosorbent assay (in-house ELISA) (Samples during the admission and convalescent phase)

Diagnostic tests to be performed in cases suspected with dengue

Dengue IgM in-house ELISA

Diagnostic tests to be performed in cases suspected with leptospirosis

Culture with Korthof's medium

LAMP and real time PCR

Diagnostic tests to be performed in cases suspected with diphtheria

Throat or nasopharynx swab is taken from the suspected patient.

5% sheep blood agar and selective Hoyle`s agar with potassium tellurite (Oxoid, Cheshire, England)

Tox-gene PCR of throat or nasopharynx swab samples (Direct toxin gene detection on the throat swabs.)

Diagnostic tests to be performed in cases suspected with meningococcal disease

Blood (buffy coat) PCR

Diagnostic tests to be performed in cases suspected with pneumonia

Chest X-ray

Sputum culture

Diagnostic tests to be performed in cases suspected with urinary tract infection

Urine culture

Diagnostic tests to be performed when an attending physician requests

Other microbiological tests, blood urea nitrogen, creatinine, AST, ALT

Laboratory procedure of each test

Blood culture

Trained nurse collected blood from a single peripheral site. We performed blood culture processing, isolate identification, and antimicrobial susceptibility testing (AST) at the SLH-Nagasaki collaborative laboratory in SLH. Blood was inoculated into two aerobic blood culture bottles of automated blood culture systems. Only aerobic culture was performed but not anaerobic culture. We used BacT/ALERT automated system (Organon-Teknika Corp., Durham, N.C.) between Jun 2015 and November 2017. The blood culture bottles were BacT/ALERT FA Plus adult bottle for patients aged 7 years or more and BacT/ALERT PF Plus pediatric bottle for patients aged under 7 years. Since December 2017, we have used BACTEC 9050 system (Becton Deckinson, Franklin Lakes, NJ). BACTEC aerobic bottle for patients aged 7 years or more and BACTEC Peds Plus for patients aged under 7 years. Blood culture bottles were assessed by comparing the weight before and after inoculation. All culture bottles were incubated for 5 days. Bottles flagging positive were sub-cultured in Columbia sheep blood, chocolate, and MacConkey's agars. The isolate identification was performed by MALDI Biotyper (Bruker Daltonics, Bremen, Germany) with additional standard microbiological techniques and VITEC2 compact (bioMérieux, France). Optochin susceptibility test was used to distinguish *Streptococcus pneumoniae* from other alpha hemolytic streptococcus. Organisms which were considered contaminated by physician and microbiologist were excluded from the analysis. These organisms are frequently associated with contamination including coagulase negative staphylococci, viridans group streptococci, *Corynebacterium* spp., *Bacillus* spp., *Micrococcus* spp. and *Propionibacterium* spp. AST was done by modified Kirby-Bauer disc

diffusion method using Sensi-Disc (BD: Becton, Dickinson and Company, USA) with additional VITEC2 compact according to the Clinical and Laboratory Standards Institute guidelines[1]. Other clinical samples such as sputum and urine were processed by standard microbiological methods.

If MALDI-TOF identification was *Burkholderia thailandensis or B. pseumomallei, Salmonella enterica* serotype Paratyphi A*, or S. enterica* serotype Typhi*,* DNA was extracted using QIAamp DNA Blood Mini Kit following the manufacturer's instructions and PCR identification was applied using primers described elsewhere [2–4]. We also performed *Salmonella* somatic and flagellar serotyping antisera tests to confirm *S.* Paratyphi A and *S.* Typhi according to the manufacturer's instructions; (Denka Sieken, Japan).

Diagnostic tests for dengue

Dengue RDT

Dengue RDTs were performed according to the manufacturers' instructions using plasma sample. Dengue Rapid diagnostic test (RDT) (Dengue Duo, NS1, IgM and IgG, Standard Diagnostics, South Korea).

Dengue virus Conventional RT-PCR [5]

RNAs were extracted from 100 μL plasm portion by using ISOGEN II (Wako, Japan) accor ding to the manufacturer's protocol. The generic pan-dengue primers used, which targeted th e 3′ noncoding region of dengue viruses, were pan-dengue forward (5′- TCAATATGCTGAA ACGCGCGAGAAACCG-3′) and pan-dengue reverse (5′- GAAAACTTTTCTTCGTACCACGG GACTAA -3′). Conventional RT-PCR reactions were performed on the Veriti Thermal Cycler (Applied Biosystems, US) using PrimeScript One Step RT-PCR Kit Ver.2 (Takara, Japan). Cy cling conditions were the following: 50°C for 30 min and 94°C for 2 min then 30 cycles o f 94°C for 30 sec, 56°C for 30 sec and 72°C for 1 min. Capillary electrophoresis system, MultiNA (Shimadzu Corporation, Japan), was used to detect the PCR product.

In-house anti-dengue IgM capture ELISA [6]

Plasma samples were sent to Research Biotechnology Division, St Luke's Medical Center. In-house anti-dengue IgM ELISA was performed. The samples were diluted in 1:100. Optical densities were determined based on absorbance readings, and then the positive - negative ratio (P/N ratio) was calculated. A cut-off value of 2.00 was used based on the result of the negative control population.

Leptospirosis diagnostic test

Whole cell-based IgM ELISA (Patoc-IgM ELISA)

The *Leptospira biflexa* serovar Patoc antigen coating plate was prepared according to the WHO guidance [7]. Excess binding sites of the well were blocked with 200 μl per well of 20 mg/ml of BSA in 20 mM Tris, 150 mM NaCl, 0.05% Tween 20, pH 7.5 (TBST) for 1.5 h at room temperature (RT), after which the BSA solution was removed. The plate was rinsed six times with 200 μl per

well of distilled water, and then blocked with 200 μl per well of 20 mg/ml of BSA in TBST for 1.5 h at RT. The plate was then rinsed three times with 300 μl per well of TBST. 50 μl of patient plasma samples diluted 400-fold with ELISA buffer were added in a total volume of 50 μl per well and incubated for 1.5 h at RT. The plasma was then rinsed four times with 200 μl per well of TBST then replaced with 50 μl per well of peroxidase-conjugated goat anti-human IgM solution (QED Bioscience) diluted 5000-fold with ELISA buffer and then incubated for 1 h at RT. The goat anti-human IgM solution was then rinsed out as above. Finally, 50 μl of o-phenylenediamine dihydrochloride solution (OPD tablet (Sigma) in 6 ml distilled, deionized water containing 0.02% hydrogen peroxide) was added and settled for 2 min, and the reaction was stopped by adding 50 μl per well of 1M sulfuric acid solution. Absorbance at 492 nm of each well was quantitated in a microtiter-plate reader (Biotek Epoch- Mircroplate spectrophotometer / microplate reader). The mean+3 standard deviation (SD) value of blood donor controls were defined as the cut-off limit for a positive result. Positive for Leptospirosis Patoc-IgM ELISA were defined as positive results of Patoc-IgM ELISA with diluted 400-fold plasma of admission samples or convalescent samples.

Leptospirosis blood culture

Blood culture was performed using Korthof's medium. After the sample collection, 1–2 drops of blood were put into Korthof's medium and cultivated at 30°C up to 13 weeks. The cultures were examined weekly by dark-field microscopy. Positive cultures were identified by MAT and PCR.

Leptospirosis blood (plasma) PCR

Extracted DNA from plasma samples were used for PCR of leptospirosis. Lepto-*rrs* LAMP was used as screening when the patients were suspected with leptospirosis. When the Lepto-*rrs* LAMP was positive, we performed real time PCR as confirmation. Patients with positive for both PCR tests were determined as PCR positive leptospirosis

Lepto-*rrs* LAMP

Lepto-*rrs* LAMP was performed using previously described primers and conditions [8]. The r eaction mixture (25 μl) for the Lepto-*rrs* LAMP contained 1.6 μM each primer (FIP, 5′-TAG TTTCAAGTGCAGGCTGCGAGGCGGACATGTAAGTCAGG-3′; BIP, 5′-GGAGTTTGGGAGA GGCAAGTGGGCCACTGGTGTTCCTCCA-3′; LF, 5′-GTTGAGCCCGCAGTTTTCAC-3′; LB, 5′-AATTCCAGGTGTAGCGGTGA-3′) and 0.2 μM other primers (F3, 5′-TCATTGGGCGTAA AGGGTG-3′; B3, 5′-AGTTTTAGGCCAGCAAGTCG-3′), in addition to 20 mM Tris-HCl (pH 8.8), 10 mM KCl, 8 mM $MgSO_4$, 10 mM $(NH_4)_2SO_4$, 0.1% Tween 20, 0.8 M betaine, 0.7 2 mM each deoxynucleotide triphosphate, 1 μl of a fluorescent detection reagent (Eiken Che mical Company, Tochigi, Japan), 8 U of *Bst* DNA polymerase (Lucigen, Middleton, WI), and

2 μl of DNA template. DNA templates were heated to 95°C for 2 min, followed by rapid cooling on ice before addition to the LAMP reaction mixture. LAMP reactions were perform ed at 65°C for 60 min, followed by termination at 95°C for 5 min using Veriti Thermal Cy cler (Applied Biosystems, US). Positive and negative results were distinguished by UV fluor escence.

Leptospirosis real time PCR blood (plasma)

Primers and a probe that target leptospiral 16S ribosomal RNA gene (*rrs*) were designed for a real-time PCR assay {F3C (400 nM), 5′-TCATTGGGCGTAAAGGGTG-3′; B3C (600 nM), 5′-TCAGTTTTAGGCCAGCAAGTC-3′; Probe (250 nM), 56-FAM/ AGAGGCAAG/ZEN/TGGAATTCCAGGTG/BHQ}. Real time PCR reactions were performed on the StepOne (Applied Biosystems, US) using the One Step PrimeScript RT-PCR Kit (Takara, Japan). Results were evaluated on the linear scale with slope correction and a threshold of 0.05. A positive result was considered any exponential curve with a cycle threshold (CT) prior to cycle 35. Cycling conditions were the following: 50°C for 2 min and 95°C for 20 sec then 45 cycles of 95°C for 1 sec and 60°C for 30 sec.

Diagnostic test for diphtheria

Laboratory-confirmed cases were defined as a patient with a positive result of culture for *C. diphtheriae* and/or a tox-gene PCR positive result from throat or nasopharynx swab samples. Throat or nasopharynx swab specimens were taken at the time of admission from all patients with clinically suspected diphtheria. Brain heart infusion (BHI) broth was used as transport medium. The swabs were inoculated to 5% sheep blood agar and selective Hoyle′s agar with potassium tellurite (Oxoid, Cheshire, England). Black suspect colonies on Hoyle′s agar plate which were Gram-positive bacilli were identified using MALDI Biotyper (Bruker Daltonics, Bremen, Germany). The Minimum Inhibitory Concentrations (MICs) of penicillin G and erythromycin were determined by E-test strips according to manufacturer’s instruction (bioMérieux, Lyon, France). We assessed the toxigenicity of the identified isolates by PCR that detects the tox gene using a published method [9]. Direct toxin gene detection was also performed on the throat swabs. DNA was extracted from the 100 μL aliquot of the BHI transport broth using a QIAamp DNA Blood Mini Kit following manufacturer's instruction (QIAGEN, Hilden, Germany).

Diagnostic tests for meningococcal disease [10,11]

The genes can be targeted in *Neisseria meningitidis* species-specific assays, *ctrA* and *sodC*. The capsule transport to cell surface gene, *ctrA*, is highly conserved among isolates responsible for invasive meningococcal infections and has been used in both real-time and conventional PCR to

detect *N. meningitidis*. Primers of *ctrA* F753 (5′-GCTGCGGTAGGTGGTTCAA-3′) and R (5′-TTGTCGCGGATTTGCAACTA-3′). Extracted DNA from buffy coat was used for the *ctrA* PCR to detect *N. meningitidis*. Conventional PCR reactions were performed on the Veriti Thermal Cycler (Applied Biosystems, US) using *TaKaRa Ex Taq* Hot start Version (Takara, Japan). Cycling conditions were the following: 94°C for 2 min then 35 cycles of 94°C for 30 sec, 56°C for 30 sec and 72°C for 1 min. Capillary electrophoresis system, MultiNA (Shimadzu Corporation, Japan), was used to detect the PCR product. Capsular group determination was performed using conventional PCR. The sequences shown in Table 2 were the primers used following the standardized protocol by the World Health Organization & Centers for Disease Control and Prevention (2011). The presence of the target capsular gene determines the corresponding capsular group of the *N. meningitidis* isolate. Conventional serogrouping PCR assay for genotyping for serogroup was performed using extracted DNA from blood buffy coat samples or isolates when blood PCR or blood culture was positive following the WHO manuals [10]. Because preliminary analysis showed serogroup B and Y were common in Manila, we used the primers of serogroup B (*synD*) and Y(*synF*).

Diagnostic tests of X-ray

If pneumonia was suspected, Chest X-ray was ordered by an attending physician. The X-ray was assessed by both radiologist and the physician by blind method. They were requested to choose three categories namely, "not pneumonia", "possible pneumonia", and "highly suspected pneumonia". If both the radiologist and the physician chose "highly suspected pneumonia", we defined the patients as X-ray confirmed pneumonia.

CBC, Procalcitonin (PCT), C Reactive Protein (CRP)

CBC parameters were evaluated using an automated hematology analyzer (Sysmex XN-1000, Sysmex, Kobe, Japan). The Rapidchip PCT kit (Sekisui Medical, Japan) was used to measure PCT. The detection of PCT was based on immunochromatography technique using Quantitative Immunoassay Analyzer (Sekisui Medical, Japan). The Alere Afinion CRP kit with Alere Afinion AS100 Analyzer (Alere Medical, Japan) was used to measure CRP. Tests require 120 μl and 1.5 μl of either serum or whole blood and have a detection range of 0.2-10 ng/ml and 5-160 mg/L, respectively.

Analysis of *Staphylococcus aureus*

Laboratory test for MRSA was done by the cefoxitin disk diffusion test. Detection of inducible clindamycin resistance was by the disc approximation D-zone test. In addition, PCR and multiplex PCR of the 16S rDNA for *S. aureus*, *mecA* gene, PVL(*lukF-lukS*) and SCCmec types were performed on all *S. aureus* strains [12,13]. SCCmec types (type I, IA, II, III, IV, IVA) were determined with the multiplex PCR. These types are commonly identified in South Asian countries.

DNA extracted from isolates was stored at −20°C using a DNA extraction kit (Wizard Genomic DNA Purification Kit; Promega) and transported to Nagasaki, Japan for multilocus sequence typing (MLST) as described by Bolt et al [14]. The MLST of *S. aureus* uses seven housekeeping genes combinations of *arcC, aroE, glpF, gmk pta, tpi* and *ygiL*. Sequence types (STs) were assigned by the PubMLST database and added to this database (https:// pubmlst.org /). We generated a phylogenetic tree by using Molecular Evolutionary Genetics Analysis (MEGA) software version MEGA 7.0.26 (https://www.megasoftware.net/).

Flow of making final diagnosis:

In case more than two laboratory tests were positive, final diagnosis was made following the Supplementary table1. The following case definitions were used. A bacteremia group was defined as positive blood culture if organisms were not considered contaminated. A proven dengue virus infection was defined as positive result of NS1 RDT or dengue RT-PCR. A probable dengue virus infection was defined as (Dengue RDT IgM positive or Dengue IgM-ELISA positive) and all other tests were negative. A proven leptospirosis are defined i) PCR or Culture positive or ii) seroconversion of Patoc-IgM ELISA. A probable leptospirosis was defined as positive results of Patoc-IgM ELISA of acute phase sample. Diphtheria infection was defined as culture positive or PCR positive of throat/nasopharynx swab. Blood culture negative meningococcal infection was defined as a negative result of blood culture and positive results of blood PCR. X-ray confirmed pneumonia group was defined as a negative result of blood culture and chest X ray showed highly suspect pneumonia. A severe skin infection was defined as a negative result of blood culture and cellulitis or possible abscess formation of the deep dermis and subcutaneous tissue.

Reference

1. Clinical & Laboratory Standards Institute (CLSI). M100Ed31 | Performance Standards for Antimicrobial Susceptibility Testing, 31st Edition. In: Clinical & Laboratory Standards Institute (CLSI) [Internet]. 2013 [cited 31 Mar 2021]. Available: https://clsi.org/standards/products/microbiology/documents/m100/

2. Ravan H, Yazdanparast R. Loop region-specific oligonucleotide probes for loop-mediated isothermal amplification-enzyme-linked immunosorbent assay truly minimize the instrument needed for detection process. Anal Biochem. 2013;439: 102–108. doi:10.1016/j.ab.2013.04.014

3. Fan F, Du P, Kan B, Yan M. The Development and Evaluation of a Loop-Mediated Isothermal Amplification Method for the Rapid Detection of Salmonella enterica serovar Typhi. PLoS One. 2015;10: e0124507. doi:10.1371/journal.pone.0124507

4. Tuanyok A, Auerbach RK, Brettin TS, Bruce DC, Munk AC, Detter JC, et al. A horizontal gene

transfer event defines two distinct groups within Burkholderia pseudomallei that have dissimilar geographic distributions. J Bacteriol. 2007;189: 9044–9049. doi:10.1128/JB.01264-07

5. National Institute of Infectious Disease in Japan. Laboratory manuals for pathogen detection, dengue fever. [cited 31 Mar 2021]. Available: https://www.niid.go.jp/niid/en/examination.html

6. Bundo K, Igarashi A. Antibody-capture ELISA for detection of immunoglobulin M antibodies in sera from Japanese encephalitis and dengue hemorrhagic fever patients. J Virol Methods. 1985;11: 15–22. doi:10.1016/0166-0934(85)90120-x

7. World Health Organization. Human leptospirosis : guidance for diagnosis, surveillance and control. 2003. Available: https://apps.who.int/iris/handle/10665/42667

8. Koizumi N, Nakajima C, Harunari T, Tanikawa T, Tokiwa T, Uchimura E, et al. A new loop-mediated isothermal amplification method for rapid, simple, and sensitive detection of Leptospira spp. in urine. J Clin Microbiol. 2012;50: 2072–2074. doi:10.1128/JCM.00481-12

9. Nakao H, Popovic T. Development of a direct PCR assay for detection of the diphtheria toxin gene. J Clin Microbiol. 1997;35: 1651–1655. doi:10.1128/JCM.35.7.1651-1655.1997

10. World Health Organization, Centers for Disease Control and Prevention (U.S.). Laboratory methods for the diagnosis of meningitis caused by neisseria meningitidis, streptococcus pneumoniae, and haemophilus influenzae : WHO manual. 2nd ed. 2011. Available: https://apps.who.int/iris/handle/10665/70765

11. National Institute of Infectious Disease in Japan. Laboratory manuals for pathogen detection, Neisseria meningitidis. [cited 31 Mar 2021]. Available: https://www.niid.go.jp/niid/en/examination/9493-labmanual-en.html

12. Oliveira DC, de Lencastre H. Multiplex PCR strategy for rapid identification of structural types and variants of the mec element in methicillin-resistant Staphylococcus aureus. Antimicrob Agents Chemother. 2002;46: 2155–2161. doi:10.1128/aac.46.7.2155-2161.2002

13. Lina G, Piémont Y, Godail-Gamot F, Bes M, Peter MO, Gauduchon V, et al. Involvement of Panton-Valentine leukocidin-producing Staphylococcus aureus in primary skin infections and pneumonia. Clin Infect Dis. 1999;29: 1128–1132. doi:10.1086/313461

14. Bolt F, Cassiday P, Tondella ML, Dezoysa A, Efstratiou A, Sing A, et al. Multilocus sequence typing identifies evidence for recombination and two distinct lineages of Corynebacterium

diphtheriae. J Clin Microbiol. 2010;48: 4177–4185. doi:10.1128/JCM.00274-10

Appendix 2.

Table S1. Final laboratory and clinical confirmed diagnosis when more than two pathogens were identified by study tests

Laboratory test (N=)	Total number of detected	Blood culture(+)	Dengue NS1 (+) or RT-PCR (+)	Dengue {NS1(-) and RT-PCR(-)} and {RDT IgM(+) or ELISA IgM(+)}	Lepto PCR(+) or Culture(+) or Lepto IgM seroconversion	{Lepto PCR(-) Culture(-)} and IgM (+)	X ray confirmed pneumonia	Diphtheria PCR(+) or culture (+)	Meningococcus PCR(+)	Severe Skin infection (+)
Blood culture (+)	77		B (0)	B (1)	B (2)	B (3)	B (8)	N (0)	B (8)	B (15)
Dengue NS 1(+) or RT-PCR (+)	79			D (0)	N (3)	D (2)	D (2)	Diph (0)	M (0)	N (0)
Dengue {NS1 (-) and RT-PCR (-)} and {RDT IgM (+) or ELISA IgM (+)}	80				L (9)	N (5)	N (5)	Diph (2)	M (2)	N (3)
Lepto PCR (+) or Culture (+) or Lepto IgM seroconversion	97					L (0)	L (2)	N (0)	N (1)	L (0)
{Lepto PCR (-) or Culture (-)} and IgM (+)	45						N (2)	Diph (0)	M (0)	N (0)
X ray confirmed pneumonia	86							Diph (1)	M (0)	N (2)
Diphtheria PCR (+) or culture (+)	50								N (0)	Diph (0)
Meningococcus PCR (+)	26									M (0)
Severe kin infection (+)	59									

N:No laboratory diagnosis, B: Bacteremia, D:proven Dengue: D, pD: probable Dengue, L: proven leptospirosis, pL: probable leptospirosis, P: Xray confirmed pneumonia, Diph: Diphtheria, M: Meningococcus, S: Severe skin infection

Appendix 3.

Table S2. Characteristics of patients enrolled as the dengue control (Clinical diagnosed with dengue fever)

		Clinically diagnosed with Dengue fever (N=257)	%
Enrolled season	2015July~2016Jun	0	0
	2016July~2017Jun	47	18.3
	2017July~2018Jun	98	38.1
	2018July~2019Jun	112	43.6
Dry season (Nov~Jun)		180	70
Rainy season (July~Oct)		77	30
Age group	Under 5	7	2.7
	6- 17y	93	36.2
	18 years and above	157	61.1
Sex	Female	99	38.5
	Male	158	61.5
Place of residence (1missing data)	Manila City	96	37.4
	Outside Manila City	161	62.7
Duration of fever(1missing data)	<=7 days	247	96.1
	>7days	10	3.9
Underlying chronic condition N (%)(1missing value)	No	220	86.3
	Yes	35	13.7
Antibiotic use before admission	No	199	77
	Yes	59	23
Referral from other facilities	No	179	69.7
	Yes	78	30.4
BT 37> on admission	No	104	40.5
	Yes	153	59.5
Shock (mean blood pressure < 70)	No	247	96.1
	Yes	10	3.9
Glasgow Coma Scale	=15	257	100
	< 15	0	0
qSOFA	<2	249	96.9
	>=2	8	3.1
WBC× 10^9/L (19 missing value)	<15.0	252	98.1
	>=15.0	5	2
Neutrophils % (18 missing value)	<80	242	94.2
	>=80	15	5.8

CRP (10> mg/dL)(8missing value)	<10	242	94.2
	>=10	15	5.8
PCT (0.75> ng/mL)(47 missing value)	<0.75	148	59.7
	>=0.75	100	40.3
Blood culture positive		0	0
Mortality, n (%)		0	0

CRP, C-reactive protein; PCT, procalcitonin; qSOFA, quick Sequential Organ Failure Assessment;

Appendix 4.

Table S3. Physical and clinical signs of enrolled patients and the association with positive results of blood culture

	N. of showing the signs	% / enrolled patients	N.of BC positive	BC positive ratio	P value	Odds ratio(95% confidence interval)
Presenting symptoms						
Headache	880	66.9%	55	6.3	0.39	1.25 (0.75~2.08)
Rigor	289	22.0%	19	6.6	0.56	1.17 (0.69~2.01)
Cough or sputum	734	55.8%	41	5.6	0.64	0.90 (0.56~1.42)
Dyspnea	559	42.5%	38	6.8	0.21	1.34 (0.85~2.13)
Bloody diarrhea	50	3.8%	2	4.0	0.57	0.66 (0.16~2.77)
Abdominal pain	677	51.5%	45	6.7	0.21	1.35 (0.85~2.15)
Coma or confusion	70	5.3%	8	11.4	0.04	2.2 (1.01~4.77)
Joint pain	654	49.7%	47	7.2	0.04	1.63 (1.02~2.61)
Rash during illness	341	25.9%	23	6.7	0.42	1.23 (0.74~2.04)
Wound	236	17.9%	21	8.9	0.03	1.78 (1.06~3.01)
Sore throat	428	32.5%	24	5.6	0.79	0.93 (0.57~1.53)
Chest pain	344	26.2%	29	8.4	0.02	1.77 (1.10~2.86)
Water diarrhea	396	30.1%	16	4.0	0.07	0.59 (0.34~1.04)
Vomiting or nausea	710	54.0%	33	4.7	0.04	0.62 (0.39~0.98)
Physical examination by attending physician						
Anaemia	110	8.4%	7	6.4	0.81	1.10 (0.49~2.46)
Jaundice	131	10.0%	10	7.6	0.36	1.38 (0.69~2.75)
Conjunctival suffusion	228	17.3%	9	4.0	0.18	0.62 (0.30~1.25)
Rale	307	23.3%	16	5.2	0.58	0.85 (0.48~1.50)
Murmur	12	0.9%	2	16.7	0.11	3.31 (0.71~15.40)
Abdominal tenderness	458	34.8%	31	6.8	0.3	1.28 (0.80~2.05)
Hepatomegaly	21	1.6%	4	19.1	<0.01	3.94 (1.29~12.00)
Costovertebral angle tenderness	162	12.3%	14	8.6	0.11	1.64 (0.89~2.99)
Edema	44	3.3%	6	13.6	0.03	2.66 (1.09~6.52)
Skin congestion	87	6.6%	6	6.9	0.67	1.21 (0.51~2.56)
Rash	280	21.3%	18	6.4	0.60	1.16 (0.67~2.00)
Lymph adenopathy	148	11.3%	5	3.4	0.17	0.53 (0.21~1.34)
Joint swelling	20	1.5%	5	25	<0.01	5.66 (2.00~16.01)
Calf tenderness	169	12.9%	8	4.7	0.53	0.79 (0.37~1.67)
Neck stiffness	60	4.6%	8	13.3	0.01	2.64 (1.21~5.79)

Appendix 5.

Table S4. The blood culture volume and blood culture positivity

Table S4-1. The definition of adequacy for the blood culture volume

	Recommend Volume	Adequate > = 80%	Less adequate 80% - 50%	Underfilled less than 50%
over 12 years	10ml×2	>=16ml	8ml~15.9ml	below 8ml
4-11 years	5ml×2	>=8ml	4ml~7.9ml	below 4 ml
1-3 years	3ml×2	>=4.8ml	2.4ml~4.9ml	below 2.4 ml

Table S4-2. The blood culture positivity and the number of blood culture volume*

	Negative	Positive	BC Positivity	P value
1 Bottles	211	11	4.95	0.256
2 Bottle	728	25	3.32	

Table S4-3. The blood culture positivity and the adequacy of the blood volume for blood culture*

	Negative	Positive	BC Positivity	P value
Adequate >= -20%	424	14	3.2	0.601
Less adequate -20% to -50%	339	13	3.69	
Underfilled less than 50%	176	9	4.86	

*N=947, 367 missing values due to unavailable to weight the blood culture bottle
