# Supplementary material for: A prospective observational study of community-acquired bacterial bloodstream infections in Metro Manila, the Philippines
Source: PLoS Negl Trop Dis. 2022 May 25;16(5):e0010414. doi: 10.1371/journal.pntd.0010414 (PMC9173634; doi:10.1371/journal.pntd.0010414)
Supplement: S2 File — (PDF) [file pntd.0010414.s002.pdf]

STROBE Statement—checklist of items that should be included in reports of observational studies

A prospective observational study of community-acquired bacterial bloodstream infections in Metro Manila, the Philippines

	Item No.	Recommendation	Page No.	Relevant text from manuscript
**Title and abstract**	1	(*a*) Indicate the study's design with a commonly used term in the title or the abstract	P1	A prospective observational study of community-acquired bacterial bloodstream infections in Metro Manila, the Philippines
		(*b*) Provide in the abstract an informative and balanced summary of what was done and what was found	P3~ P5	
**Introduction**				
Background/rationale	2	Explain the scientific background and rationale for the investigation being reported	P6~ P7	L76 ~ L92
Objectives	3	State specific objectives, including any prespecified hypotheses	P6 ~ P7	The objective of this study was to provide contemporary data concerning the aetiology and AMR patterns in patients admitted to hospital in Metro Manila with a suspected community acquired bacterial bloodstream infection.
**Methods**				
Study design	4	Present key elements of study design early in the paper	P 7 ~ P13	
Setting	5	Describe the setting, locations, and relevant dates, including periods of recruitment, exposure, follow-up, and data collection	P7	L100~L108
Participants	6	(*a*) *Cohort study*—Give the eligibility criteria, and the sources and methods of selection of participants. Describe methods of follow-up<br>*Case-control study*—Give the eligibility criteria, and the sources and methods of case ascertainment and control selection. Give the rationale for the choice of cases and controls<br>*Cross-sectional study*—Give the eligibility criteria, and the sources and methods of selection of participants	P 8 ~P9 Additional information is described in Supplementary materials	L111 ~ L135
		(*b*) *Cohort study*—For matched studies, give matching criteria and number of exposed and unexposed		

		Case-control study—For matched studies, give matching criteria and the number of controls per case		
Variables	7	Clearly define all outcomes, exposures, predictors, potential confounders, and effect modifiers. Give diagnostic criteria, if applicable	Not applicable	
Data sources/ measurement	8*	For each variable of interest, give sources of data and details of methods of assessment (measurement). Describe comparability of assessment methods if there is more than one group	P12	L186 ~ L192
Bias	9	Describe any efforts to address potential sources of bias	P 20	Not explained in the method but explained in the discussion<br>L328 ~ L 340
Study size	10	Explain how the study size was arrived at	Not applicable	Not explained in the manuscript and the sample size was not determined because the study aims to determine aetiologies of bacterial blood stream infection among all eligible patients in one single health facility in certain period.

Continued on next page

Quantitative variables	11	Explain how quantitative variables were handled in the analyses. If applicable, describe which groupings were chosen and why	P12	L186 ~ L192
Statistical methods	12	(*a*) Describe all statistical methods, including those used to control for confounding	P12	L186 ~ L192
		(*b*) Describe any methods used to examine subgroups and interactions	Not applicable	
		(*c*) Explain how missing data were addressed	Not written in manuscript	There were small number of missing data and the method addressing in missing data explained in table foot note
		(*d*) *Cohort study*—If applicable, explain how loss to follow-up was addressed<br>*Case-control study*—If applicable, explain how matching of cases and controls was addressed<br>*Cross-sectional study*—If applicable, describe analytical methods taking account of sampling strategy	Not applicable	
		(*e*) Describe any sensitivity analyses	Not applicable	
**Results**				
Participants	13*	(a) Report numbers of individuals at each stage of study—eg numbers potentially eligible, examined for eligibility, confirmed eligible, included in the study, completing follow-up, and analysed	P13<br>Figure1<br>Supplementary material	L203 ~ L212
		(b) Give reasons for non-participation at each stage	P13<br>Figure1<br>Supplementary material	L203 ~ L212
		(c) Consider use of a flow diagram	Figure1	
Descriptive data	14*	(a) Give characteristics of study participants (eg demographic, clinical, social) and information on exposures and potential confounders	P13~P14	L214~L226
		(b) Indicate number of participants with missing data for each variable of interest	Table1	
		(c) *Cohort study*—Summarise follow-up time (eg, average and total amount)	Not applicable	
Outcome data	15*	*Cohort study*—Report numbers of outcome events or summary measures over time	Not applicable	
		Case-control study—Report numbers in each exposure category, or summary measures of exposure		
		Cross-sectional study—Report numbers of outcome events or summary measures		

Main results	16	(*a*) Give unadjusted estimates and, if applicable, confounder-adjusted estimates and their precision (eg, 95% confidence interval). Make clear which confounders were adjusted for and why they were included	Not applicable	
		(*b*) Report category boundaries when continuous variables were categorized	Table 1	Age group were categorized.
		(*c*) If relevant, consider translating estimates of relative risk into absolute risk for a meaningful time period	Not applicable	

Continued on next page

Other analyses	17	Report other analyses done—eg analyses of subgroups and interactions, and sensitivity analyses	Not applicable	
**Discussion**				
Key results	18	Summarise key results with reference to study objectives	P17	L277 ~ L285
Limitations	19	Discuss limitations of the study, taking into account sources of potential bias or imprecision. Discuss both direction and magnitude of any potential bias	P20	L 328 ~ L 340
Interpretation	20	Give a cautious overall interpretation of results considering objectives, limitations, multiplicity of analyses, results from similar studies, and other relevant evidence	P17<br>Table 5	L 281 ~ L 283<br>L296 ~ L 299
Generalisability	21	Discuss the generalisability (external validity) of the study results	P20	L 335 ~ L 338
**Other information**				
Funding	22	Give the source of funding and the role of the funders for the present study and, if applicable, for the original study on which the present article is based	P22	L368 ~ L370

*Give information separately for cases and controls in case-control studies and, if applicable, for exposed and unexposed groups in cohort and cross-sectional studies.

Note: An Explanation and Elaboration article discusses each checklist item and gives methodological background and published examples of transparent reporting. The STROBE checklist is best used in conjunction with this article (freely available on the Web sites of PLoS Medicine at http://www.plosmedicine.org/, Annals of Internal Medicine at http://www.annals.org/, and Epidemiology at http://www.epidem.com/). Information on the STROBE Initiative is available at www.strobe-statement.org.
