# Supplementary material for: A prospective observational study of community-acquired bacterial bloodstream infections in Metro Manila, the Philippines
Source: PLoS Negl Trop Dis. 2022 May 25;16(5):e0010414. doi: 10.1371/journal.pntd.0010414 (PMC9173634; doi:10.1371/journal.pntd.0010414)
Supplement: Attachment — Submitted filename: response_CAB_plos ntd_20220323.docx [file pntd.0010414.s004.docx]

24^th^ March 2022

*Shaden Kamhawi*

*Editor-in-Chief*

*PLOS Neglected Tropical Diseases*

Dear Editor:

Thank you very much for giving us the opportunity to revise and resubmit our manuscript (Manuscript Number: PNTD-D-21-01281) titled ““A prospective observational study of community-acquired bacterial bloodstream infections in Metro Manila, the Philippines.” We are very grateful for your encouraging review of our manuscript.

The manuscript has been rechecked and revised and the necessary changes have been made in accordance with the reviewers’ suggestions. The responses to all comments have been prepared and attached herewith.

We believe that the reviewers’ comments and suggestions have greatly improved our manuscript, and we hope that the revised manuscript is now suitable for publication in *PLOS Neglected Tropical Diseases.*

The corresponding author for this paper is Koya Ariyoshi and the First Author is Nobuo Saito

We look forward to hearing from you at your earliest convenience.

Sincerely,

Koya Ariyoshi, MD, PhD (corresponding author)

School of Tropical Medicine and Global Health, Nagasaki University, Nagasaki, Japan

Department of Clinical Medicine, Institute of Tropical Medicine, Nagasaki University, Japan

Sakamoto 1-12-4, Nagasaki 852-8523, Japan

E-mail: kari@nagasaki-u.ac.jp

Telephone: +81-(0)95-819-7842

Fax: +81-(0)95-819-7843

Nobuo Saito MD, MSc, PhD

School of Tropical Medicine and Global Health, Nagasaki University, Nagasaki, Japan

1-12-4 Sakamoto Nagasaki 852-8523, Japan

E-:mail: nobuosaito@oita-u.ac.jp

Telephone: +81-(0)95-819-7008

Fax: +81-(0)95-819-7583

**Comments and response Reviewer 1**

Dear Reviewer

We are grateful for the reviewer’s critical comments and useful suggestions that have helped us improve our paper. As indicated in the responses below, we have considered all the reviewer’s comments and suggestions when preparing the revised version of our paper. All revisions are highlighted in the revised manuscript. We thank the reviewer for the kind comments.

Comments

- Reviewer comment #1:

*Overall, I think the study presents some useful data, and the authors should be commended on the amount of work it must have taken to collect the information.  My main concerns relate to a clearer articulation of the hypothesis and presentation of the analysis, but publication would be worthwhile if these can be addressed.*

Response to comment #1

The main aim of this study was to identify the etiology and AMR patterns of bacterial bloodstream infections through systematic sample collection of blood culture and other tests in a prospective study in the Philippines. The other aim was to determine the proportion of blood cultures that were positive and the characteristics of patients with positive blood cultures in a dengue endemic area. Our hypothesis was that there is the difference in the clinical and laboratory characteristics between the patients with positive blood culture and negative blood culture. We modified the structure and sentences in the introduction accordingly. We added sentences in abstract (L40~46) and introduction (L96 ~ L100, L106 ~ L109).

- Comment #2:

*While this was mainly a descriptive study of patients with bloodstream infection, and the objectives are explained, there are no hypotheses clearly stated. Yet, statistical tests with p values etc are presented in the results, so hypothesis testing is implied. I think these should be more clearly articulated or it should be clarified that this was a purely descriptive study and no hypotheses are being examined. However, there are clearly some hypotheses being tested here - eg. whether BC positivity changes over time, by seasons, by clinical variables etc*

Response to comment#2

We have now articulated the study hypotheses in the manuscript. Please see Response to comment #1

- Comment #3

*Yes, the design is broadly appropriate.  I think the choice of control group may need some further explanation.  Why only dengue positive cases were chosen, for instance? Why this sample size for the control population, or was it just a convenience sample?  It seems the point of the control group was to demonstrate that positive blood cultures are not being missed in patients with other febrile syndromes*

Response to comment #3

We appreciate the reviewer`s comments. We understood that we failed to explain the important point in our manuscript. Among febrile patients admitting to this hospital, in 30~40% the patients are diagnosed with dengue infection. We wished to determine if the clinical diagnosis of dengue fever was reliable and that bacterial blood stream infection were not being missed. A convenience sample was recruited for this purpose as explained in method section (L144~L150).

We also emphasize the importance of dengue fever as the differential diagnosis of bacteremia or BSI in the abstract (L40~43), introduction (L95~97), method and discussion (L404~407).

- Comment #4

*(editor comment) Ln 116: What does “clinically suspected to have a bacterial bloodstream infection” mean exactly?*

Response to comment #4

We appreciate this comment. Patients were recruited to the study when the admitting clinicians and study team considered, based on the clinical presentation and clinical features, that they may have a bacterial blood stream infection and as a result a blood culture was requested by the attending physician. We added the sentence in L132~136. “When the attending physician and study team suspected the patient to have bacteraemia and therefore requested a blood culture this was defined as “clinically suspected to have a community-acquired bacterial bloodstream infection”.”

- Comment #5

*No formal sample size calculations are presented - and this ties back to the lack of clear hypotheses. While the sample size is probably reasonable for this kind of descriptive study, questions of power adequacy are hard to answer as it is not clear what precisely is being tested.*

Response to comment #5

One of our aims was to determine the proportion of significant positive blood cultures. We added this explanation in the method section (L211~L215). *“We assumed that the proportion of significant positive blood cultures would be about 8% [4]. We planned to recruit 1250 patients which would result in 100 patients with a significant bacteraemia. We considered this would be a reasonable sample to understand the range of pathogens causing bacteraemia and to compare the clinical features of those with a positive blood culture with those that were negative.*” We also add sentences about our limitation to analyze clinical characteristics and parameters among patients with a positive blood culture compared with those with a negative blood culture by statistical methods (L392~398).

- Comment #6

*a) I am not sure that ORs to compare the year of admission (using 2015-6 as a baseline reference) and the rate of BC positivity is the best way to do this.  I would have thought that a test for temporal trends might be more appropriate (although I am not a statistician so seeking advice on this would be recommended).  I wonder whether it might be more informative to present these graphically in some way, which may also show seasonal variation as well as year-year trends?*

Response to #6

The aim of this analysis was to assess whether there was a significantly higher proportion of blood cultures in a certain year. However, as the reviewer has suggested, we have removed this analysis because of the short duration of the study (only 4 years) and the small sample size in each month.

Comment #7

*b) there are mulitple ORs calculated for a number of clinical and demographic variables - was there any adjustment for multiplicity of testing?  Is there not a risk that some associations will thrown up by chance alone?*

Response to #7

We did not perform any adjustment for the multiplicity of testing. It is possible that significant differences may have occurred by chance due to multiple comparisons. We have explained this as a limitation in discussion (L392~398). *“We identified several clinical signs and blood results that were associated with a positive blood culture. Of note, the sample size (n=1315) had an adequate power (>80%) if there was more than a 12% difference in the presence of a parameter between the blood culture positive and a negative groups. Our analysis is limited because we did not correct for multiple comparisons and did not perform multivariable analysis to adjust ORs. Further analysis is necessary to identify features that indicate in which patients it would be useful to take a blood culture.”*

Comment #8

*c) It was a little unclear with the "initial" clinical diagnosis if several of these were made on the basis of the blood culture results (e.g. meningococcus, endocarditis), in which case comparing the likelihood of BC positivity seems confounded and not very informative (and again comparing ORs to pneumonia as a baseline condition seems like an unusual way to present things)?*

Response to # 8

We changed the term of “initial diagnosis” into “admission diagnosis” and added an explanation in the methods section (L135~138). *“The most likely diagnosis was selected from our diagnostic lists as an admission diagnosis by an attending physician during the enrolment. The admission diagnoses were made clinically in the ER before laboratory tests were available.”*

The admission diagnosis was made before taking blood culture. For example, a clinical diagnosis of meningococcal disease were often made due to the presence of a typical skin rash. We agree, calculating the OR and p-values in comparison to pneumonia is not appropriate and has been removed from Table1.

- Comment #9

*The presentation of multiple ORs in Table 1 is not really explained or justified in the statistical methods (see above)*

Response to #9

We explained it in method section (L217~222). *“To identify statistical difference between patients with a positive blood culture and those with a negative result, categorical variables were compared with chi-squared test or Fisher’s exact test, and numerical variables by the Student’s t test. To investigate the effect of various variables on the blood culture positivity, an unadjusted logistic regression model was used to show odds ratio.”*

- Comment #10

*-Are the results clearly and completely presented?*

*Otherwise OK - see above about presentation and analysis of the seasonal / time trends*

*They state that no MDR/XDR E coli strains were found, but at least one was meropenem resistant in Table 3 - are the authors sure there was no carbapenem-resistant E coli, which would normally be at least MDR if not XDR? Some further clarity here would be useful.*

Response to #10

We wrongly used the data of one isolate of study ID 1605. We repeated the antimicrobial susceptibility testing by disc test and VITEK®2 methods for this isolate and it was found to be susceptible to meropenem. We have revised manuscript (Table 3) and supplementary file (data sheet, S3_File).

- Comment #11

*-Are the limitations of analysis clearly described?*

*I think it would be worth stating that Leptospira IgM positivity alone (or even seroconversion with confirmatory testing like MAT) can have false positives.*

Response to #11

We added the sentence accordingly. (L410~412) *” We used IgM Leptospirosis ELISA as a confirmatory test for proven or probable leptospirosis, although the test can cause false positive results [41].”*

- Comment #12

*Table 5: check spelling of species as some errors e.g. "Acinetoaceter", "B. pseudomalle" "S pneumonia"*

Response to #12

We appreciate the reviewer`s comments. We modified accordingly in the Table 5.

- Comment #13

*Author summary "Melioidosis" is a disease, not a pathogen - suggest "Burkholderia pseudomallei" is referring to the organism*

Response to #13

We modified accordingly in the abstract (L52) and the discussion (L420).
